# Phytochemical Profiling, Antioxidant Capacity, and In Vitro α-Amylase/β-Glucosidase Inhibitory Activity of Different Avocado Tissues Under Methanolic and CO_2_-Assisted Aqueous Extraction

**DOI:** 10.3390/molecules31193418

**Published:** 2026-09-25

**Authors:** Elif Apaydın

**Affiliations:** Central Research Laboratory Application and Research Center, Giresun University, 28200 Giresun, Türkiye; elif.apaydin@giresun.edu.tr; Tel.: +90-543-5873941

**Keywords:** avocado by-products, *Persea americana*, seed coat, green extraction, polyphenols, antioxidant capacity, enzyme inhibition

## Abstract

The study evaluated the phenolic profile, antioxidant capacity, and in vitro α-amylase and fungal β-glucosidase inhibitory activity of avocado fruit pulp, peel, cotyledon, and seed coat (testa) extracts obtained using methanol and a CO_2_-assisted aqueous extraction method. Total phenolic content (TPC) and total flavonoid content (TFC) were determined spectrophotometrically; antioxidant activity was assessed by DPPH, CUPRAC, and TEAC assays; and individual phenolic compounds were quantified by a validated HPLC-UV method. Enzyme inhibitory activity against α-amylase and β-glucosidase was measured in vitro, and IC_50_ values were calculated and compared with acarbose as a reference standard. Methanolic extracts generally showed higher TPC, TFC, and higher concentrations of several HPLC-quantified phenolics per gram of extract than the CO_2_-water extracts. All extracts inhibited both α-amylase and β-glucosidase, yielding IC_50_ values in the low mg/mL range. Inhibitory potency was highest in the seed coat extract in both assays, while the ordering of peel and cotyledon differed slightly depending on the enzyme considered. Avocado peel and seed coat, particularly in methanolic extracts, showed relatively high phenolic content, antioxidant capacity, and mild in vitro enzyme inhibition; however, these findings reflect glycosidase screening only and require further validation in cell-based and in vivo metabolic models.

## 1. Introduction

Avocado (*Persea americana* Mill.) is a widely consumed fruit from the Lauraceae family. The industrial processing of avocados generates substantial by-products, mainly peel and seed tissues, which can account for approximately 20–30% of the fruit’s mass. These by-products are increasingly recognised as potential sources of value-added bioactive compounds [1,2,3]. In addition to the edible pulp, avocado tissues contain a variety of phytochemicals, including phenolic acids, flavonoids, carotenoids, and related antioxidant metabolites. The composition of these phytochemicals varies significantly among anatomical fractions, cultivars, maturity stages, and growing conditions [1,2,3]. Recent profiling studies consistently show that peel and seed fractions are richer in polyphenols than pulp, although the dominant fraction differs by cultivar and assay system [2,3,4,5]. Advanced chromatographic analyses have identified broad phenolic diversity in avocado by-products, including gallic, chlorogenic, caffeic, ferulic, and *p*-coumaric acids together with quercetin derivatives, catechins, and procyanidins, supporting their interest for food, nutraceutical, and cosmetic applications [2,3].

The recovery of these compounds depends strongly on the extraction method and solvent system. Conventional alcoholic solvents remain highly effective for phenolic extraction from avocado matrices, and several studies report that methanolic or ethanolic systems yield higher phenolic recovery and stronger antioxidant responses than more aqueous systems [1,6]. At the same time, there is growing interest in green extraction methods for fruit by-products, because extraction conditions can selectively enrich different phenolic classes rather than simply maximizing total yield [7,8,9,10]. In the context of avocado and other plant matrices, the efficiency of extraction is influenced by a number of factors. These include solvent polarity, pressure, temperature, co-solvent composition, and matrix structure. Greener extraction systems have been observed to demonstrate efficacy in the extraction of certain compounds, while exhibiting reduced effectiveness in the extraction of other compounds [7,8,9]. This makes direct comparison between a conventional solvent and a greener alternative especially relevant for avocado by-products, where the targeted compounds and intended applications can differ substantially across tissues [3,8,11].

Plant phenolics and flavonoids are also widely studied for their capacity to inhibit carbohydrate-digesting enzymes linked to postprandial glucose release, particularly α-amylase, but the evidence from cell-free assays should be interpreted as preliminary rather than as proof of antidiabetic efficacy [11,12]. Large reviews show that flavonoid-rich extracts and purified flavonoids frequently inhibit α-amylase and related digestive enzymes in vitro, yet translation beyond enzyme assays requires caution because activity depends on compound structure, assay design, and biological context [11,13]. In avocado specifically, peel and seed residues have already been described as phenolic-rich antioxidant materials, and recent work has emphasized their broader valorization potential, but direct side-by-side comparison of pulp, peel, seed, and isolated seed coat under both methanolic and CO_2_-assisted aqueous extraction remains limited [3,4]. Accordingly, the present study compares the phytochemical profiles, antioxidant capacity, and in vitro α-amylase/β-glucosidase inhibitory activities of avocado fruit pulp, peel, seed, and seed coat obtained using methanolic and CO_2_-assisted aqueous extraction, with particular emphasis on the underexplored seed coat as a distinct by-product fraction.

## 2. Results and Discussion

### 2.1. Biomass Distribution and Extraction Yields

The mass balance and extraction efficiency of the four distinct anatomical fractions of avocado fruit (*Persea americana*) were evaluated using both green CO_2_-assisted aqueous extraction and conventional methanolic extraction (Table 1).

The natural biomass yield scaled per 1 kg of fresh fruit demonstrated that the mesocarp (fruit pulp) constitutes the vast majority of the fruit mass (715.3 g/kg), followed by the exocarp (peel) (170.57 g/kg) and cotyledon (seed kernel) (111.37 g/kg). On the other hand, the seed coat (or testa) constitutes a small anatomical fraction, yielding a maximum of 2.75 g/kg of fresh biomass (Table 1).

With regard to recovery performance, extraction yields differed significantly among anatomical parts and the applied parameters.

Seed Coat (Testa): As demonstrated in Table 1, the highest crude extraction yields were exhibited in both protocols, reaching 28.50% (2.8 g extract/10.00 g sample) with methanolic extraction and 23.0% (1.15 g extract/5.00 g sample) with CO_2_-assisted aqueous extraction. This elevated yield is attributed to the high density of soluble polyphenolic compounds and proanthocyanidins concentrated within the testa layer.

Exocarp (Peel): The yield of crude extract under methanolic conditions was 24.10%, in comparison to 18.80% obtained using the CO_2_-water protocol (Table 1). The increased duration of the extraction process, which extended to eight hours, in conjunction with the enhanced solubility of methanol, resulted in a substantial recovery of mid-polar flavonoids from the external peel layer.

Cotyledon (Seed Kernel): As demonstrated in Table 1, the extraction process was found to be moderately efficient, yielding 15.20% in methanol and 12.60% in CO_2_-water.

Mesocarp (Fruit Pulp): A distinct trend was exhibited, wherein the CO_2_-water protocol yielded a higher recovery (11.60%) in comparison to methanolic extraction (8.30%), as shown in Table 1. The enhanced performance of the aqueous phase in the mesocarp is likely driven by ultrasonic cavitation disruption (3 times 1 min) and a higher liquid-tosolid ratio (1:10 g/mL), which facilitated the dissolution of hydrophilic polysaccharides and organic acids present in the fruit pulp.

### 2.2. Total Phenolic and Flavonoid Content Analysis Results

The TPC and TFC values of the four avocado fractions obtained by the two extraction systems are presented in Table 2. Both tissue type and extraction protocol were associated with differences in the measured spectrophotometric values (*p* < 0.001).

Methanol extracts yielded considerably higher TPC and TFC values than the corresponding CO_2_-water extracts across all fractions. Among methanol extracts, the highest TPC was observed in peel (76.034 mg GAE/g dry extract), followed by fruit pulp (65.129 mg GAE/g dry extract), seed coat (37.114 mg GAE/g dry extract), and seed (22.615 mg GAE/g dry extract). TFC values followed a similar trend, with peel showing the highest concentration (35.042 mg QE/g dry extract) and fruit pulp a comparable value (32.352 mg QE/g dry extract); the seed had the lowest TFC (3.954 mg QE/g dry extract).

In the CO_2_-water extracts, a similar distributional pattern was observed, although all values were lower than those obtained with methanol. Peel again showed the highest TPC (41.381 mg GAE/g dry extract), followed by seed coat (33.990 mg GAE/g dry extract), fruit pulp (31.546 mg GAE/g dry extract), and seed (20.022 mg GAE/g dry extract). Flavonoid recovery was slightly reduced in CO_2_-water extracts relative to methanol; the methanol:CO_2_-water TFC ratio reached up to 10-fold for fruit pulp and peel, reflecting the limited capacity of this greener solvent system to recover moderately lipophilic flavonoids.

Methanolic extracts showed higher measured values in several assays, but the present design does not isolate solvent polarity from other protocol differences. Physiologically, the consistently high polyphenol content of the peel across both extraction systems is consistent with its role as a protective barrier accumulating secondary metabolites against pathogens and UV radiation. Notably, the seed coat surpassed the cotyledon in both TPC and TFC under both extraction conditions, indicating that the outer protective layers of avocado by-products (peel and seed coat) hold considerable valorisation potential.

### 2.3. Antioxidant Activity: DPPH, CUPRAC, and TEAC

Antioxidant results for both extraction systems are presented in Table 3 and Figure 1, a comparative summary with literature values is provided in Table 4.

#### 2.3.1. Methanol Extracts

In methanol extracts, fruit pulp exhibited the highest DPPH radical scavenging activity (158.785 ± 0.190 µmol TE/g dry extract), considerably exceeding peel (73.429 ± 0.116 µmol TE/g dry extract), seed (74.787 ± 0.180 µmol TE/g dry extract), and seed coat (64.239 ± 0.330 µmol TE/g dry extract). In contrast, peel showed the highest CUPRAC reducing power (467.776 ± 1.657 µmol TE/g dry extract), followed by fruit pulp (333.521 ± 2.102 µmol TE/g dry extract), seed (171.297 ± 0.762 µmol TE/g dry extract), and seed coat (147.405 ± 1.177µmol TE/g dry extract). TEAC values for methanol extracts were highest in fruit pulp and peel (~17.6–17.8 µmol/g dry extract), much lower in seed (5.706 ± 0.001 µmol/g dry extract), and intermediate in seed coat (15.287 ± 0.002 µmol/g dry extract).

#### 2.3.2. CO_2_-Water Extracts

Antioxidant values were generally lower in CO_2_-water extracts than in methanol extracts, consistent with the reduced phenolic recovery observed in TPC and TFC analyses [2,6,14,15]. A notable exception was the seed coat, which maintained a comparatively high TEAC value (16.478 ± 0.006 µmol/g dry extract) in CO_2_-water extracts substantially higher than the other fractions (2.138–4.377 µmol/g dry extract range) under the same extraction system. This pattern suggests assay-dependent antioxidant behavior, potentially reflecting differences in phenolic composition, but the present data do not identify the specific compounds responsible for the elevated TEAC response [4,16].

**Table 4:** Comparison of solvent and fraction effects on antioxidant assays with selected literature values for avocado by-products.

Fraction	Solvent Effect (Literature)	Antioxidant Pattern (This Study)	Citations
Pulp	Lower phenolics compared to by-products in most studies	High DPPH in MeOH; reduced in CO_2_–water	[2,16,17,18]
Peel	Often highest phenolics and DPPH/ABTS/TEAC	Highest CUPRAC in MeOH; moderate TEAC	[16,17,18,19]
Seed	Frequently strong DPPH and FRAP with optimized ethanol/water	Moderate DPPH/CUPRAC; very low TEAC, especially CO_2_–water	[4,20,21]
Seed coat	Reported strong antioxidant activity in electron/hydrogen transfer assays (FRAP/ABTS)	Moderate DPPH/CUPRAC, but consistently high TEAC, even with CO_2_–water	[18,22,23]

The present study confirms the high antioxidant capacity of the peel in a reducing-power assay (CUPRAC), in agreement with reports that peel is frequently the richest avocado fraction in total phenolics and FRAP/TEAC [16,17,18,19]. Previous studies have characterised seed coat phenolics using advanced hyphenated MS methods [22,23] but have not directly compared this fraction against pulp, peel, and cotyledon using multiple classical antioxidant assays in parallel. And although a strong reducing agent such as ascorbic acid represents a thermodynamically superior reference standard for CUPRAC measurements due to its standard reduction potential, Trolox was selected in this study to maintain consistent equivalence units (µmol TE/g extract) across the multi-assay panel (DPPH, CUPRAC, and TEAC). This standardization allows direct relative comparisons among tissue fractions, though the adoption of ascorbic acid as the primary reducing standard is recognized as the preferred approach for standalone CUPRAC evaluations in future research. The observation that seed coat TEAC is unusually high and is retained in CO_2_-water extracts provides new evidence for the distinct phenolic composition of this fraction, which may reflect uncharacterized constituents not covered by the eight analyte HPLC system [4,18].

### 2.4. HPLC Analysis of Individual Phenolic Compounds

The validated HPLC method achieved baseline separation of all eight target phenolic analytes within a single 75 min run (Figure 2). Most target compounds showed high linearity; however, caffeic acid should be reported separately because its reported R^2^ was 0.9939 and acceptable peak tailing factors (0.942–1.354), with LOD and LOQ values ranging from 0.005 to 0.018 mg/mL and 0.011 to 0.052 mg/mL, respectively. Precision was satisfactory, with RSD values below 1.72% for all analytes (Table 5). Full analytical validation including spike-recovery assays for accuracy, matrix effect evaluation, intermediate precision (inter-day variability), and method robustness was not performed. Consequently, the quantitative data presented should be interpreted as comparative estimates across the tested extraction protocols rather than as absolute, fully validated quantifications.

The quantitative distribution of phenolic compounds in the avocado extracts is presented in Table 6. Methanolic extracts contained quantifiable levels of more target analytes in most tissues, whereas many analytes in CO_2_-water extracts were reported as ND or <LOD. Among methanol extracts, fruit pulp exhibited the highest abundance of phenolic acids, with chlorogenic acid (4.216 ± 0.002 mg/g dry extract) and ferulic acid (2.656 ± 0.003 mg/g dry extract) being the most abundant. Gallic acid (2.236 ± 0.006 mg/g dry extract), caffeic acid (1.155 ± 0.001 mg/g dry extract), p-coumaric acid (1.025 ± 0.001 mg/g dry extract), syringic acid (0.954 ± 0.002 mg/g dry extract), trans-cinnamic acid (1.247 ± 0.002 mg/g dry extract), and quercetin (0.523 ± 0.001 mg/g dry extract) were also quantifiable in fruit pulp methanol extracts.

In contrast, CO_2_-water extraction yielded substantially reduced phenolic concentrations, with many compounds falling below the limit of detection (<LOD) or not detected (ND) in peel, seed, and seed coat fractions. In the reported Table 6, quercetin was not detected in all CO_2_-water extracts, and this should be described consistently in the text.

The HPLC profile quantified in this study comprising gallic acid, chlorogenic acid, caffeic acid, syringic acid, p-coumaric acid, ferulic acid, trans-cinnamic acid, and quercetin is consistent with the main phenolic acids and flavonoids reported in avocado peel, pulp, and seeds by previous studies [24,25,26]. Hydroxycinnamic acid derivatives (chlorogenic, p-coumaric, ferulic, and caffeic acid) dominate peel and seed profiles in the literature [24,26,27], hydroxybenzoic acids (gallic and syringic acid) are part of reported peel and seed profiles [24,28] and quercetin the flavonol marker included in the present panel is widely detected in avocado by-products alongside catechin/epicatechin and procyanidins [4,17]. The detailed separate profiling of the seed coat in the present study is consistent with advanced studies reporting 42–52 individual phenolics in avocado seed and seed coat fractions, dominated by phenolic acids and flavonoids [22]; condensed tannins, catechins, epicatechins, and procyanidins which may contribute substantially to the high TPC of this fraction were not directly quantified in the present work and represent an important gap for future investigation.

### 2.5. Enzyme Inhibition: α-Amylase and β-Glucosidase

All avocado extracts inhibited both α-amylase and β-glucosidase in vitro, yielding IC_50_ values in the low mg/mL range (Table 7). Methanolic extracts generally showed lower IC_50_ values than CO_2_-water extracts, but the current dataset does not establish a direct causal relationship between bulk TPC/TFC and enzyme inhibition. Among all fractions, the seed coat methanol extract showed the lowest IC_50_ values for α-amylase (1.25 mg/mL) and β-glucosidase (1.89 mg/mL), followed by peel and cotyledon (with nearly equivalent IC_50_ values of 2.20 and 2.25 mg/mL, respectively), and fruit pulp.

It must be emphasised that acarbose, the reference inhibitor, exhibited lower IC_50_ values than all plant extracts under the same in vitro conditions: 0.86 mg/mL for α-amylase and 1.18 mg/mL for β-glucosidase. Therefore, all extracts tested are less potent than acarbose as assessed in this enzyme inhibition model. This finding is consistent with the results reported by Chokki et al. (2020) [29], who observed that Morinda lucida and Momordica charantia leaf extracts inhibited both enzymes in the low mg/mL range, although acarbose remained more potent than the plant extracts. Nonetheless, the IC_50_ values recorded here are consistent with the low mg/mL range commonly reported for phenolic-rich plant extracts in equivalent in vitro assay systems, and have been associated with biologically relevant antidiabetic activity in numerous studies [11,30,31]. Extrapolation of these in vitro findings to antidiabetic efficacy in vivo must be approached with caution, as factors including bioavailability, metabolic transformation, and physiological concentrations at the intestinal level are not reflected by IC_50_ values measured under cell-free, single-enzyme conditions.

Across the assay conditions used here, all extracts produced lower IC_50_ values against α-amylase than against β-glucosidase. Because the two enzymes were assayed using different substrates, temperatures, and reaction conditions, this pattern should not be interpreted as biochemical selectivity. This selectivity pattern has been previously reported for hydroxycinnamic acid derivatives and flavonoids present in avocado by-products [13,32].

A notably interesting finding was the strong inhibitory activity of CO_2_-water seed coat extract against α-amylase (IC_50_ = 1.65 mg/mL), which closely approached that of the methanol extract (1.25 mg/mL). This proximity suggests that CO_2_-water extraction conditions may selectively co-extract compounds with α-amylase inhibitory activity independently of bulk phenolic yield, possibly reflecting differences in the polarity profile or structural characteristics of the extracted compounds rather than overall phenolic content.

The enzyme inhibitory results obtained in this study are consistent with previous reports of in vitro α-amylase inhibition by avocado-derived phenolic extracts, while the inhibitory activity observed here against β-glucosidase should be interpreted within the context of the specific assay system used in the present work. Dhakal et al. (2022) [33] reported low IC_50_ values for methanol extracts of Nepalese avocado seed against both enzymes, consistent with the present findings for seed and seed coat fractions. In this context, acarbose was used as a reference inhibitor to enable comparison with data from other studies. However, its moderate IC_50_ against fungal β-glucosidase (*A. niger*) reflects its intrinsic selectivity towards mammalian intestinal α-glucosidase and α-amylase systems rather than fungal β-glycosidic linkages. It showed that aqueous avocado seed extract exerts antidiabetic effects in alloxan-diabetic rats and that these effects are associated with enhanced PI3K/Akt signaling, evidenced by reversal of diabetes-induced reductions in PI3K and AKT expression together with improved insulin status and glucose-lipid metabolism [34]. However, direct translation from the in vitro enzyme inhibition data of the present study to such in vivo mechanisms is not appropriate, and the antidiabetic potential suggested by the IC_50_ values should be regarded as preliminary, warranting validation in cell-based models and ultimately in vivo studies.

### 2.6. Multivariate Data Analysis: Hierarchical Clustering and PCA

To explore the biochemical and functional relationships among the four avocado fractions, hierarchical cluster analysis (HCA) coupled with a heatmap was performed (Figure 3). The HCA grouped fractions based on their total phenolic and flavonoid contents (TPC, TFC), individual HPLC phenolic profiles, antioxidant capacities (DPPH, CUPRAC, TEAC), and in vitro enzyme inhibitory IC_50_ values (α-amylase and β-glucosidase).

The vertical sample dendrogram on the left identified two primary clusters. The first main cluster was formed exclusively by fruit pulp, which segregated cleanly from all by-product fractions due to its distinct phenolic profile (specifically chlorogenic, ferulic, and gallic acids) and high DPPH radical scavenging activity. The second cluster comprised the three by-product fractions (peel, seed, and seed coat), with peel forming a distinct sub-cluster characterized by the highest TPC (76.034 mg GAE/g dry extract) and CUPRAC reducing power (467.8 μmol TE/g dry extract). Seed and seed coat clustered closely together in a sister clade, sharing similar secondary metabolite profiles and enzyme inhibitory patterns, with the seed coat displaying the lowest in vitro IC_50_ values for both tested enzymes. While this multivariate grouping highlights avocado peel as the richest fraction in bulk polyphenols and electron-transfer reducing power, it also reflects the distinct, assay-specific enzyme inhibitory responsiveness of the seed and seed coat fractions, which occurs independently of total phenolic quantity.

HCA and heatmap visualization were performed using Python v3.10. Prior to clustering, all quantitative variables were scaled using Min-Max normalization (0.0 to 1.0) to standardize variables across different units. Non-detected (ND) values were imputed as half of the limit of detection (LOD/2). To align the directional trend of enzyme inhibition capacity with phenolic and antioxidant parameters, IC_50_ values were transformed into their reciprocal values (1/IC_50_) prior to normalization. Dual-axis clustering was computed based on Ward’s minimum variance method using squared Euclidean distance as the measure of similarity.

Principal Component Analysis (PCA) was performed to visualize the discrimination among avocado fractions and extraction solvents. Prior to PCA, all quantitative dataset parameters were standardized via Z-score autoscaling (mean = 0, SD = 1) based on the correlation matrix to eliminate unit scaling bias. The analysis incorporated spectrophotometric TPC and TFC, antioxidant capacities (DPPH, CUPRAC, and TEAC), and enzyme inhibition parameters (α-amylase IC_50_ and β-glucosidase IC_50_). Individual HPLC phenolic analyte concentrations were excluded from this specific PCA matrix to prevent multicollinearity and focus on global bioactivity trends. Figure 4 displays both sample scores and variable component loadings along PC1 (50.5% variance) and PC2 (33.8% variance).

Methanolic extracts of fruit pulp and peel exhibited strong positive loadings along PC1, aligning closely with high total TPC, TFC, DPPH radical scavenging activity, and CUPRAC reducing power. In contrast, the α-amylase and β-glucosidase vectors were oriented along the positive PC2 axis, forming a near orthogonal (~90°) spatial arrangement relative to the TPC and TFC vectors. Pearson correlation analysis revealed no significant linear relationship between phenolic/flavonoid quantities and enzyme inhibition potency (r = 0.081, *p* = 0.849 for TPC vs. α-amylase IC_50_; r = −0.226, *p* = 0.591 for TPC vs. β-glucosidase IC_50_; r = −0.001, *p* = 0.998 for TFC vs. α-amylase IC_50_; r = −0.274, *p* = 0.512 for TFC vs. β-glucosidase IC_50_.

Formal Pearson correlation and PCA biplot analyses revealed no statistically significant correlation between bulk TPC/TFC values and enzyme inhibition (IC_50_) potencies within the present dataset. Consequently, contextual comparisons with literature reporting TPC–inhibition trends are interpreted with caution [12,30,31,35]. Furthermore, although the values for the IC_50_ of the former were consistently lower than those of the latter, this pattern is indicative of assay-specific kinetic responses rather than confirmed absolute enzyme selectivity [13,32,36,37]. Additionally, as detailed by Zhang and Tsao [38], the antioxidant action of dietary phenolics operates through co-existing hydrogen atom transfer (HAT) and single electron transfer (SET) mechanisms, depending on the solvent system, pH, and specific hydroxyl substitution patterns.

## 3. Materials and Methods

### 3.1. Chemicals and Equipment

HPLC-grade methanol and ethanol (≥99%) were procured from Carlo Erba Reagents (Milan, Italy). Gallic acid, quercetin and other analytical-grade reagents were obtained from Sigma-Aldrich (St. Louis, MO, USA). Folin–Ciocalteu reagent and 2,2-diphenyl-1-picrylhydrazyl (DPPH) were supplied by Merck (Darmstadt, Germany). Neocuproine (2,9-dimethyl-1,10-phenanthroline) was sourced from AFG Bioscience (Northbrook, IL, USA), while 2,2′-azino-bis(3-ethylbenzothiazoline-6-sulfonic acid) (ABTS) and Trolox were obtained from Isolab Chemicals (Wertheim, Germany). Other analytical-grade reagents were procured from Sigma-Aldrich (Darmstadt, Germany). α-Amylase (from *Aspergillus oryzae*, EC 3.2.1.1) and β-glucosidase (from *Aspergillus niger*, EC 3.2.1.21) and acarbose (used as the positive control in enzyme inhibition assays) were procured from Sigma-Aldrich (St. Louis, MO, USA). Absorbance measurements were performed using a Thermo Scientific Evolution Array UV-Vis spectrophotometer (Thermo Fisher Scientific, Waltham, MA, USA). Ultrapure water (18.3 MΩ·cm) was obtained from a Sartorius Stedim Arium 611UV purification system. The fungal α-Amylase and β-glucosidase from were used as in vitro screening enzymes for comparative extract evaluation and were not intended to model mammalian pancreatic α-amylase or intestinal α-glucosidase directly.

### 3.2. Plant Material and Sample Preparation

Fresh commercially available avocado fruits (*Persea americana* Mill., Hass variety; *n* = 10, approximate ripening stage: ready-to-eat/firm-ripe, purchased from a local market in [August, 2025] Giresun, Türkiye) were used in this study. The fruits were stored at a temperature of 4 °C for a maximum period of 24 h prior to processing. Prior to dissection, all fruits from the same batch were pooled to minimise inter-fruit biological variation. Each fruit was manually dissected into four distinct anatomical fractions: The four components of the seed are as follows (Figure 5): (1) the fruit pulp (mesocarp), (2) the peel (exocarp), (3) the seed coat (testa, the thin, dark-brown membrane adhering to the seed surface), and (4) the cotyledon (seed, after removal of the seed coat). The drying process was conducted under ambient conditions at a temperature of 20 °C. The fractions were left to dry in the air until they had reached a constant weight. The dried tissues were subjected to a process of fine powdering using a laboratory mill, followed by filtration through a standard sieve (0.5 mm particle size). The powdered tissues were then stored in sealed containers, with the containers being protected from light and moisture at −20 °C until extraction.

### 3.3. Extraction Methods

#### 3.3.1. CO_2_-Assisted Aqueous (CO_2_-Water) Extraction

CO_2_-assisted aqueous extraction was applied as a greener extraction alternative. For each fraction, 5.0 g of powdered material was transferred into a sealed Erlenmeyer flask (100 mL). Food-grade CO_2_ was bubbled into 50 mL of ultrapure water at a flow rate of approximately 0.5 L/min for 5 min prior to addition, yielding a slightly acidic aqueous phase (pH approximately 3.7–4.5, confirmed with a calibrated pH metre) through the formation of carbonic acid. The CO_2_-saturated water was immediately added to the powdered sample and the flask was sealed. The mixture was then sonicated in an ultrasonic bath (35 kHz) three times for 1 min each to ensure homogeneous dispersion, and subsequently shaken on an orbital shaker at 55 °C for 1 h. The sealed suspensions were maintained at +4 °C overnight to allow extended passive extraction. The extracts were filtered through Whatman No. 1 filter paper (125 mm) and the filtrate was concentrated under vacuum at 40 °C using a rotary evaporator. The resulting solid residues were stored at −20 °C until analysis [22]. The operational parameters of this protocol are summarized in Table 8.

It is evident that the specific physicochemical contribution of dissolved CO_2_ cannot be isolated independently, due to the absence of a parallel control extraction using non-carbonated water under identical physical conditions. Consequently, this procedure is evaluated as an integrated green extraction protocol rather than an isolated single variable study.

#### 3.3.2. Methanolic Extraction

For methanolic extraction, 10.0 g of each powdered fraction (fruit pulp, peel, seed, and seed coat) was combined with 50 mL of HPLC-grade methanol (100%) in a conical flask. The mixtures were placed on an orbital shaker at 40 °C for 8 h, filtered under vacuum and the filtrate was centrifuged at 5000 rpm for 20 min. The supernatants were concentrated under vacuum using a rotary evaporator at 40 °C. The resulting solid extracts were stored at −20 °C until analysis [39].

Although methanol is toxic and limits direct food-grade applications, it was selected as the conventional reference solvent to benchmark the maximum recoverable phenolic yield against the greener CO_2_-assisted aqueous system. Rotary evaporation at 40 °C removes the bulk solvent; however, trace methanol residues may persist. Consequently, the methanolic extracts generated here are intended for analytical profiling and in vitro screening rather than direct incorporation into food matrices. A food-compatible intermediate solvent such as ethanol or ethanol-water was not included in the present comparison; this is acknowledged as a limitation, and the evaluation of GRAS (Generally Recognized As Safe) solvent systems is recommended for future studies targeting industrial or nutraceutical applications.

The two extraction procedures were not identical in solid-to-solvent ratio, extraction temperature, extraction time, sonication, and post-extraction handling, so the comparison should be interpreted as a comparison between protocols rather than solvent chemistry alone. The extraction parameters of this protocol are summarized in Table 8.

### 3.4. Antioxidant Assays

#### 3.4.1. Total Phenolic and Flavonoid Contents

TPC was determined using the Folin–Ciocalteu colorimetric method and expressed as mg gallic acid equivalents per gram of dry extract (mg GAE/g dry extract). Briefly, 0.1 mL of appropriately diluted extract was mixed with 0.5 mL of diluted Folin–Ciocalteu reagent (1:10, *v*/*v*) and 0.4 mL of 7.5% (*w*/*v*) Na_2_CO_3_ solution. After incubation in the dark at room temperature for 30 min, absorbance was measured at 750 nm against a blank [40]. A gallic acid calibration curve (0.1–1.0 mg/mL) was used for quantification. It should be noted that the Folin–Ciocalteu assay is not specific to phenolic compounds and may be subject to interference from reducing sugars so that TPC values represent total reducing capacity expressed as gallic acid equivalents rather than a direct measure of phenolic purity.

#### 3.4.2. Total Flavonoid Content (TFC)

Total flavonoid content (TFC) results were calculated using a standard quercetin calibration curve prepared over a concentration TFC was determined using an aluminium nitrate colorimetric assay and expressed as mg quercetin equivalents per gram of dry extract (mg QE/g dry extract) range of 0.003–0.020 mg/mL (y = 64.318x − 0.0371, R^2^ = 0.9973, where y represents the absorbance at 415 nm and x represents the quercetin concentration in mg/mL). The reaction mixture contained 0.5 mL of extract, 0.1 mL of 10% (*w*/*v*) Al(NO_3_)_3_, 0.1 mL of 1 M sodium acetate, and 4.3 mL of 80% (*v*/*v*) ethanol. After incubation at room temperature for 40 min, absorbance was measured at 450 nm [41].

#### 3.4.3. DPPH Radical Scavenging Activity

DPPH radical scavenging activity was assessed using a 0.1 mM DPPH solution in methanol. Briefly, 100 µL of appropriately diluted extract was added to 3.9 mL of DPPH solution. After incubation in the dark at room temperature for 30 min, the decrease in absorbance was recorded at 515–517 nm [42]. Inhibition percentage was calculated as:

% INH = (A_control_ − A_sample_)/A_control_ × 100

The DPPH radical scavenging results were expressed as micromoles of Trolox equivalents per gram of dry extract (µmol TE/g dry extract). Results were calculated using a standard Trolox calibration curve prepared over a concentration range of 100–1000 µM (y = 0.0894x + 3.6377, R^2^ = 0.9904, where y represents percentage inhibition and x represents Trolox concentration in µM).

#### 3.4.4. CUPRAC Reducing Power

The cupric ion-reducing antioxidant capacity (CUPRAC) assay was determined by the reduction of the Cu^2+^-neocuproine complex to Cu^+^-neocuproine described by Apak et al. [43]. Briefly, 1.0 mL of 10 mM CuCl_2_ solution, 1.0 mL of 7.5 mM neocuproine solution in 96% ethanol), and 1.0 mL of 1.0 M ammonium acetate (NH_4_Ac) buffer (pH 7.0) were mixed in a test tube. Subsequently, 0.5 mL of extract (or Trolox standard) and 0.6 mL of distilled water were added to reach a total reaction volume of 4.1 mL. The reaction tubes were tightly sealed and incubated in the dark at room temperature for 30 min. The absorbance was measured against a reagent blank (containing all reagents except the sample/extract) at 450 nm using a UV-Vis spectrophotometer. Results were expressed as micromoles of Trolox equivalents per gram of dry extract (µmol TE/g dry extract) based on a Trolox calibration curve (40–400 µM, y = 0.0042x + 0.0264, R^2^ = 0.9979).

#### 3.4.5. TEAC (ABTS Radical Scavenging Activity)

ABTS radical cation (ABTS•^+^) was generated by reacting 7 mM ABTS solution with 2.45 mM potassium persulfate and allowing the mixture to stand in the dark at room temperature for 12–16 h. The ABTS•^+^ solution was diluted with phosphate-buffered saline (pH 7.4) to an absorbance of 0.70 ± 0.02 at 734 nm. Sample (10 µL) was added to 1 mL of ABTS•^+^ solution, and after 6 min incubation at room temperature, absorbance was recorded at 734 nm. Results were expressed as µmol Trolox equivalents per gram of extract (TEAC, µmol/g dry extract) [44].

Unless otherwise stated, all phytochemical and antioxidant results are expressed on a dry extract basis.

### 3.5. HPLC Analysis of Phenolic Compounds

Separation of eight phenolic standards (gallic acid, chlorogenic acid, caffeic acid, syringic acid, p-coumaric acid, ferulic acid, trans-cinnamic acid, and quercetin) was performed on an Agilent 1260 Infinity HPLC system equipped with a multi-wavelength UV detector set at 280 nm and 360 nm [45,46,47,48]. Separation was achieved on a GL Sciences InertSustain C18 column (5 µm, 4.6 × 250 mm) at a flow rate of 1.5 mL/min and column temperature 25 °C, and injection volume was 5 µL. The mobile phase consisted of solvent A (ultrapure water containing 3% *v*/*v* acetic acid) and solvent B (methanol). The gradient elution programme was as follows: 7–28% B (0–20 min), 28–30% B (20–35 min), isocratic at 30% B (35–50 min), 30–33% B (50–60 min), 33–42% B (60–62 min), 42–50% B (62–70 min), 50–70% B (70–73 min), followed by a 2 min re-equilibration step at 7% B. It was imperative that a one-minute stabilisation period be implemented between successive injections in order to reduce carryover effect. Quantification of individual phenolics in the extracts (mg/g dry extract) was performed by integrating chromatographic peak areas and interpolating against external calibration curves prepared from pure standards.

#### HPLC Method Validation

The HPLC method was validated for linearity, accuracy, precision, limit of detection (LOD) and limit of quantitation (LOQ) in accordance with ICH guidelines [49] and the United States Pharmacopeia [50].

Linearity was confirmed by constructing calibration curves at five concentration levels (0.05–0.18 mg/mL) for each standard, injected in triplicate. Correlation coefficients (R^2^) greater than 0.997 were accepted as evidence of satisfactory linearity. Seven of the eight analytes achieved R^2^ ≥ 0.997; caffeic acid yielded R^2^ = 0.9939, marginally below the pre-defined threshold, but was retained because all other validation parameters (precision, LOD, LOQ) remained within acceptance criteria. Precision was assessed as relative standard deviation (RSD, %) at three concentration levels (*n* = 6). LOD and LOQ were calculated from the standard deviation of the regression intercept (σ) and the slope (S) using the equations LOD = 3.3σ/S and LOQ = 10σ/S (Table 5).

### 3.6. Enzyme Inhibition Assays

The enzyme assays were designed as cell-free screening models to compare extract-level inhibitory activity under standardised in vitro conditions. It is imperative to acknowledge the inherent limitations of the study, which employed fungal α-amylase and fungal β-glucosidase. Consequently, the outcomes of the study should not be construed as direct measurements of mammalian pancreatic α-amylase inhibition or intestinal α-glucosidase inhibition.

#### 3.6.1. α-Amylase Inhibition Assay

α-Amylase inhibitory activity was evaluated in a sodium phosphate buffer (0.02 M, pH 6.9) containing 0.006 M NaCl. Each assay tube received 200 µL of α-amylase solution (0.5 mg/mL, corresponding to approximately 1.0 U/mL) and an appropriate volume (0–200 µL) of plant extract solution; the total volume was adjusted to a constant level with buffer. Mixtures were pre-incubated at 37 °C for 10 min. The enzymatic reaction was initiated by adding 200 µL of 1% (*w*/*v*) soluble starch solution and incubating at 37 °C for a further 5 min. The reaction was terminated by adding 400 µL of 3,5-dinitrosalicylic acid (DNSA) reagent, followed by heating in a boiling water bath (approximately 90 °C) for 5 min. After cooling to room temperature, samples were transferred to a 96-well microplate and absorbance was read at 540 nm. Parallel blanks without enzyme were prepared for each extract concentration to correct for background absorbance. Acarbose was used as a positive control at equivalent concentration ranges. IC_50_ values (the extract concentration yielding 50% enzyme inhibition) were determined from inhibition–concentration curves [51]. All analyses were run in triplicate.

For each concentration, four conditions were evaluated where applicable: enzyme control, sample reaction, sample blank without enzyme, and solvent control containing the highest assay concentration of the extract vehicle. The calculation of inhibition percentages was undertaken subsequent to the correction of background absorption from sample blanks.

#### 3.6.2. β-Glucosidase Inhibition Assay

β-Glucosidase inhibitory activity was assessed using p-nitrophenyl-β-D-glucopyranoside (pNPG) as substrate. Briefly, 35 µL of β-glucosidase solution (1 U/mL) was mixed with 0–70 µL of plant extract solution, and phosphate buffer (0.1 M, pH 6.9) was added to a final volume of 140 µL. Mixtures were pre-incubated at 25 °C for 10 min. Subsequently, 50 µL of 5 mM pNPG solution in the same buffer was added and incubation was continued at 25 °C for 5 min. The reaction was terminated by addition of 100 µL of 0.1 M Na_2_CO_3_ solution. The formation of p-nitrophenol (released upon substrate hydrolysis) was monitored at 405 nm. Enzyme activity measured in the absence of extract served as the 100% activity control. Acarbose was used as a positive reference inhibitor; however, it should be noted that acarbose is primarily an α-glucosidase/α-amylase inhibitor and its IC_50_ against the specific fungal β-glucosidase (*Aspergillus niger*, EC 3.2.1.21) used here may differ substantially from its clinically relevant inhibitory potency against mammalian intestinal α-glucosidase. IC_50_ values were calculated from inhibition concentration curves [52]. All analyses were run in triplicate. The activity of enzymes was defined as 100% in the absence of extract. Solvent controls containing the extract vehicle without sample were included in order to exclude vehicle-related effects on enzyme activity or on the measurement of UV absorption at 405 nm.

### 3.7. Statistical Analysis

Data are expressed as mean ± standard deviation (SD) of triplicate analyses (*n* = 3). For total phenolic content, total flavonoid content, and antioxidant assay results, statistical evaluation was performed using two-way analysis of variance (ANOVA) with extraction protocol (methanol vs. CO_2_-water) and avocado fraction (fruit pulp, peel, seed, and seed coat) as fixed factors. Main effects and protocol × fraction interaction effects were tested at a significance level of α = 0.05. When significant differences were detected, Tukey’s HSD post hoc test was applied for multiple comparisons among group means. Prior to ANOVA, normality and homogeneity of variance were assessed (PASW Statistics 18). In addition, principal component analysis (PCA) and hierarchical cluster analysis (HCA) were performed as complementary multivariate methods to explore sample grouping patterns and relationships among phytochemical and bioactivity variables, rather than to replace inferential statistical testing.

## 4. Conclusions

By integrating extraction yield data with mass balance, the high extract yield despite low biomass share that is often discarded or treated as part of the whole seed. These findings support the valorization of avocado processing by-products as sustainable sources of bioactive phenolics for the food, pharmaceutical, and cosmetic industries.

The potent in vitro α-amylase and fungal β-glucosidase inhibition observed in peel and seed coat extracts indicates that these fractions contain compounds capable of interfering with carbohydrate-hydrolyzing enzymes. However, the lack of a statistically significant correlation between bulk TPC/TFC and IC_50_ values suggests that enzyme inhibitory activity depends on specific phenolic structures or non-phenolic constituents not quantified here, rather than total phenolic content alone. The inhibitory activity against fungal β-glucosidase should be interpreted within the context of the assay system used.

The HPLC-quantified phenolic profile; gallic, chlorogenic, caffeic, syringic, p-coumaric, ferulic, and trans-cinnamic acids, along with quercetin is consistent with the hydroxycinnamic acids and flavonols reported in avocado by-product literature. The relatively high TEAC response and low IC_50_ values of the seed coat further indicate that this tissue harbors compounds with both radical-scavenging and enzyme-interacting properties.

Collectively, these results position avocado especially seed coat, followed by peel as biochemically distinct, phenolic-rich fractions with measurable in vitro bioactivity, and underscore the need for fraction-specific profiling rather than treating avocado by-products as a homogeneous matrix. Future research should prioritize the characterization of condensed tannins, catechins, and procyanidins not quantified in this study, along with biological replication and more extensive secondary metabolite characterization.

## Figures and Tables

**Figure 1:**
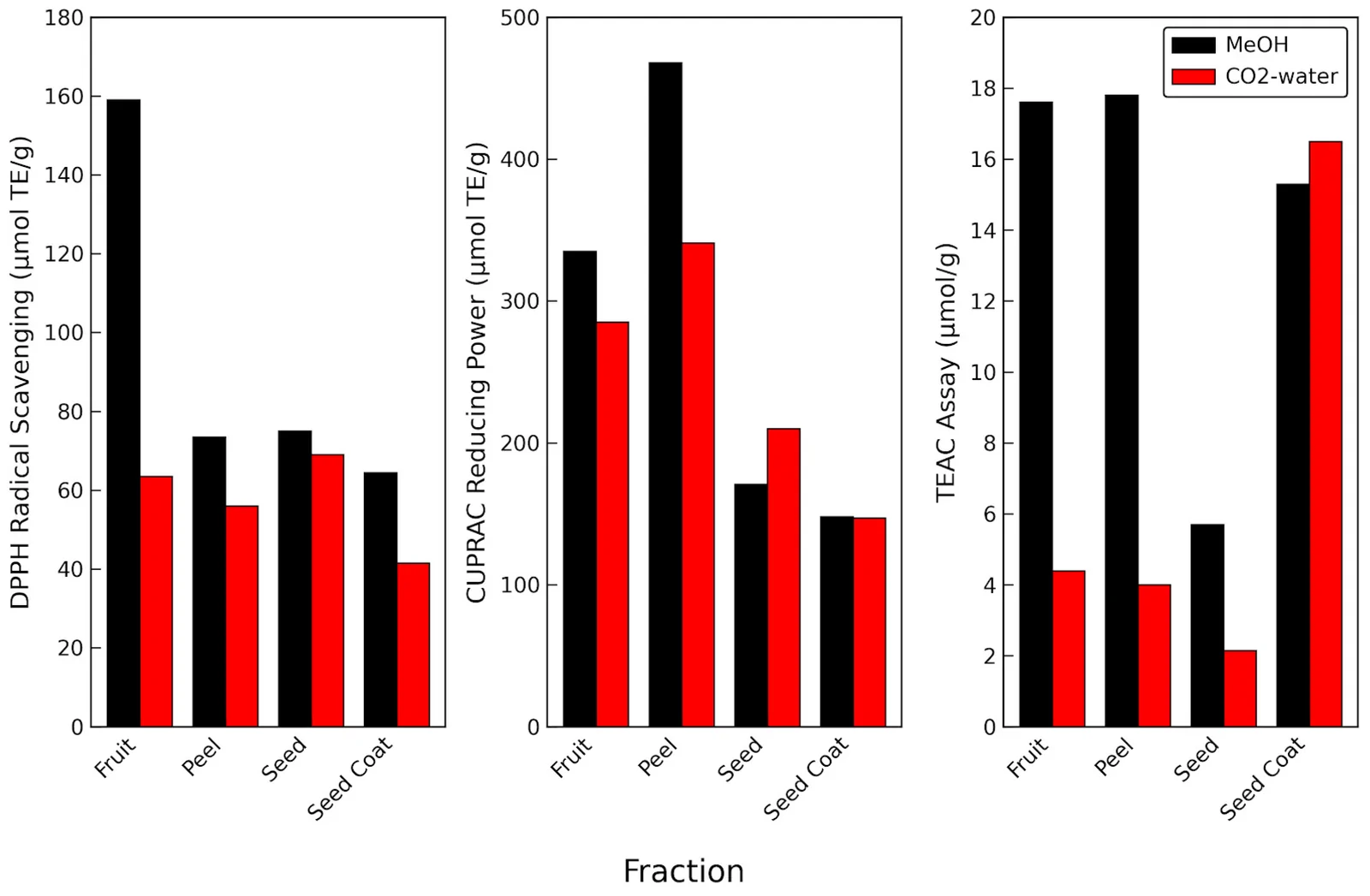
Comparative antioxidant capacities of avocado fractions (fruit pulp, peel, seed, and seed coat) extracted with MeOH and CO_2_-water across DPPH radical scavenging activity, CUPRAC reducing power, and TEAC assay (mean ± SD, *n* = 3, all values are expressed per g dry extract).

**Figure 2:**
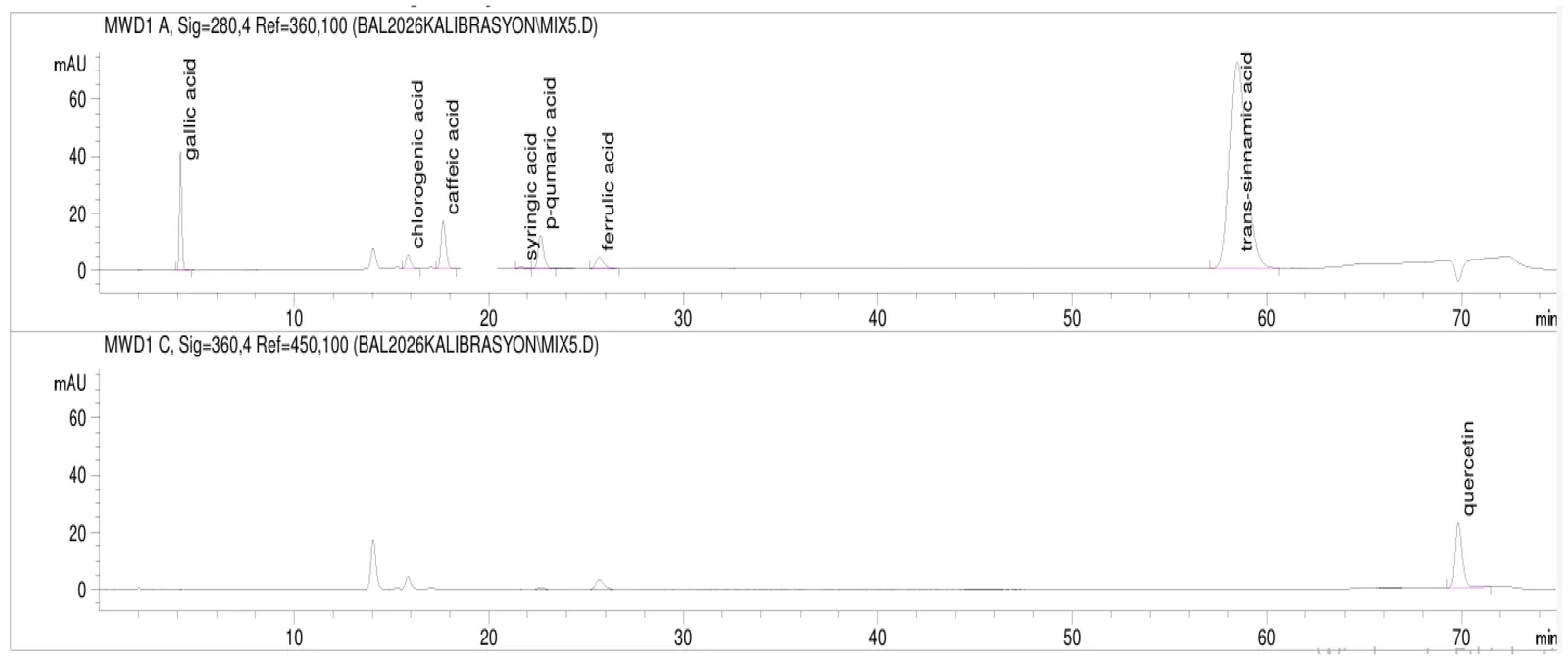
Representative HPLC chromatograms of the mixed standard solution at 280 nm (**upper panel**) and 360 nm (**lower panel**), showing baseline separation of: 1. gallic acid (RT 4.143 min); 2. chlorogenic acid (15.742 min); 3. caffeic acid (17.556 min); 4. syringic acid (21.528 min); 5. p-coumaric acid (22.489 min); 6. ferulic acid (25.458 min); 7. trans-cinnamic acid (58.834 min); 8. quercetin (70.113 min).

**Figure 3:**
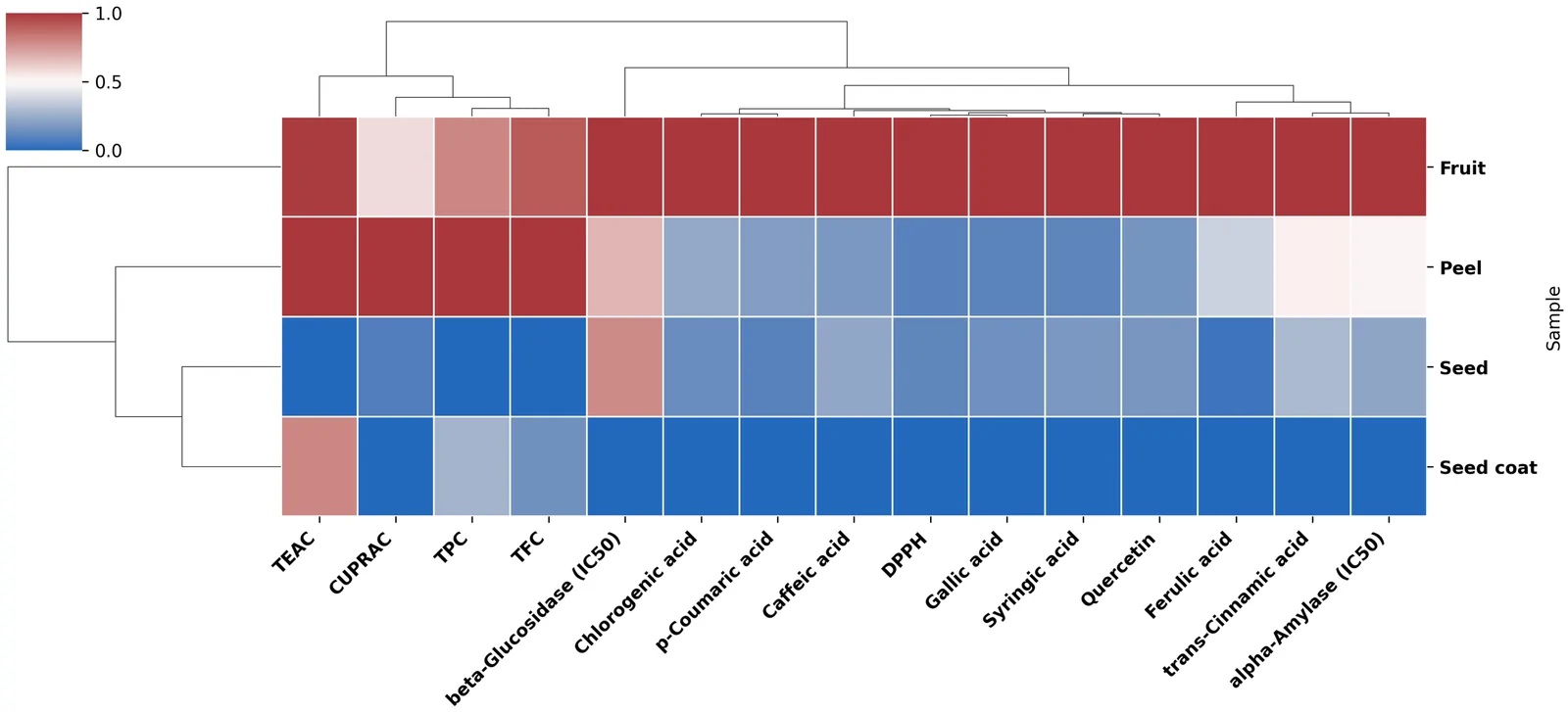
Heatmap and hierarchical cluster analysis (HCA; Ward’s method, squared Euclidean distance) of different avocado fractions based on their HPLC phenolic profiles, antioxidant capacities, and enzyme inhibitory IC_50_ values.

**Figure 4:**
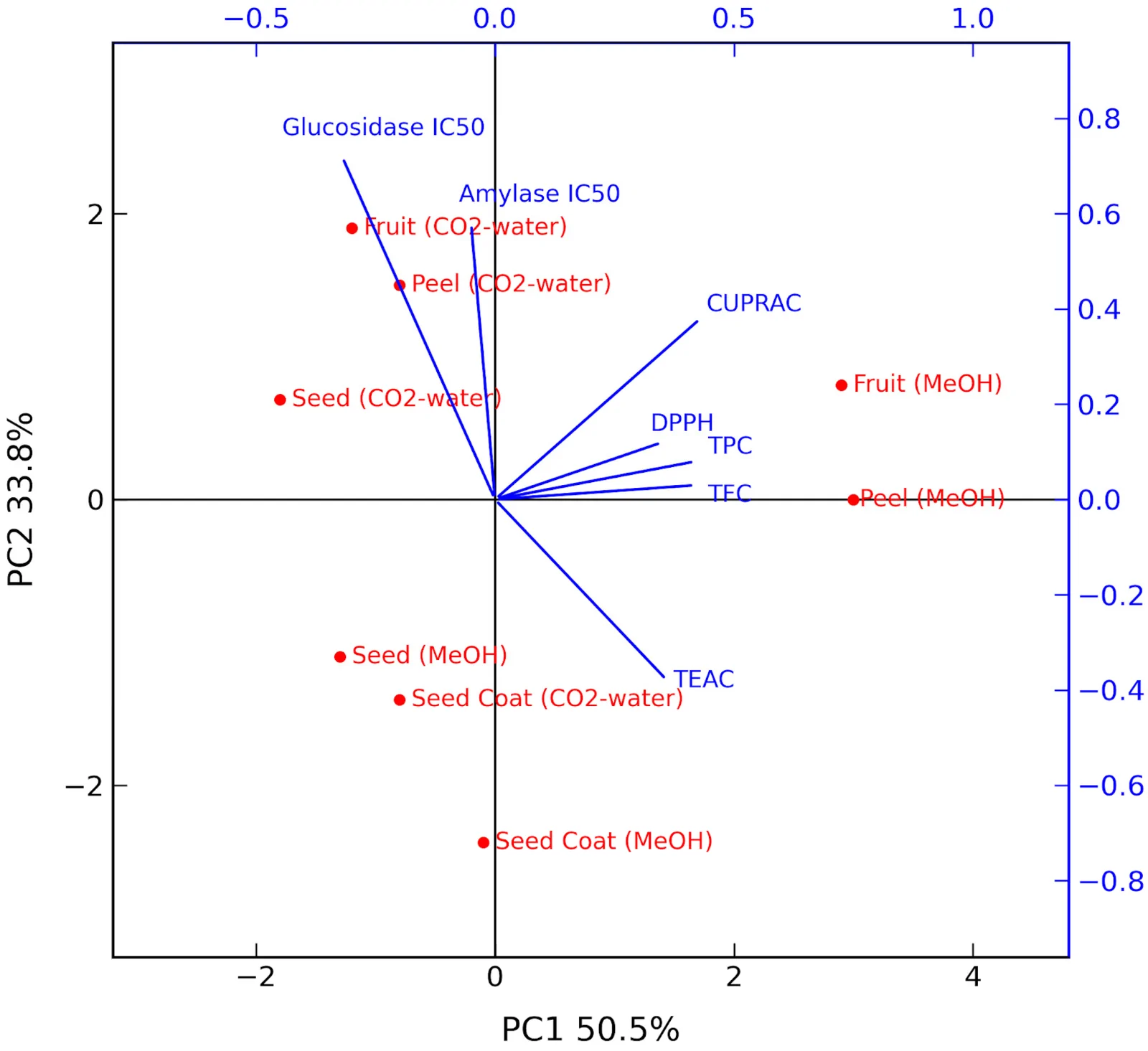
Principal Component Analysis (PCA) biplot demonstrating the clustering of avocado (*Persea americana* Mill.) anatomical fractions and extraction solvents based on their phytochemical content (TPC, TFC) and antioxidant activity profiles (DPPH, CUPRAC, TEAC) and enzyme inhibition capacities (α-amylase IC_50_ and β-glucosidase IC_50_).

**Figure 5:**
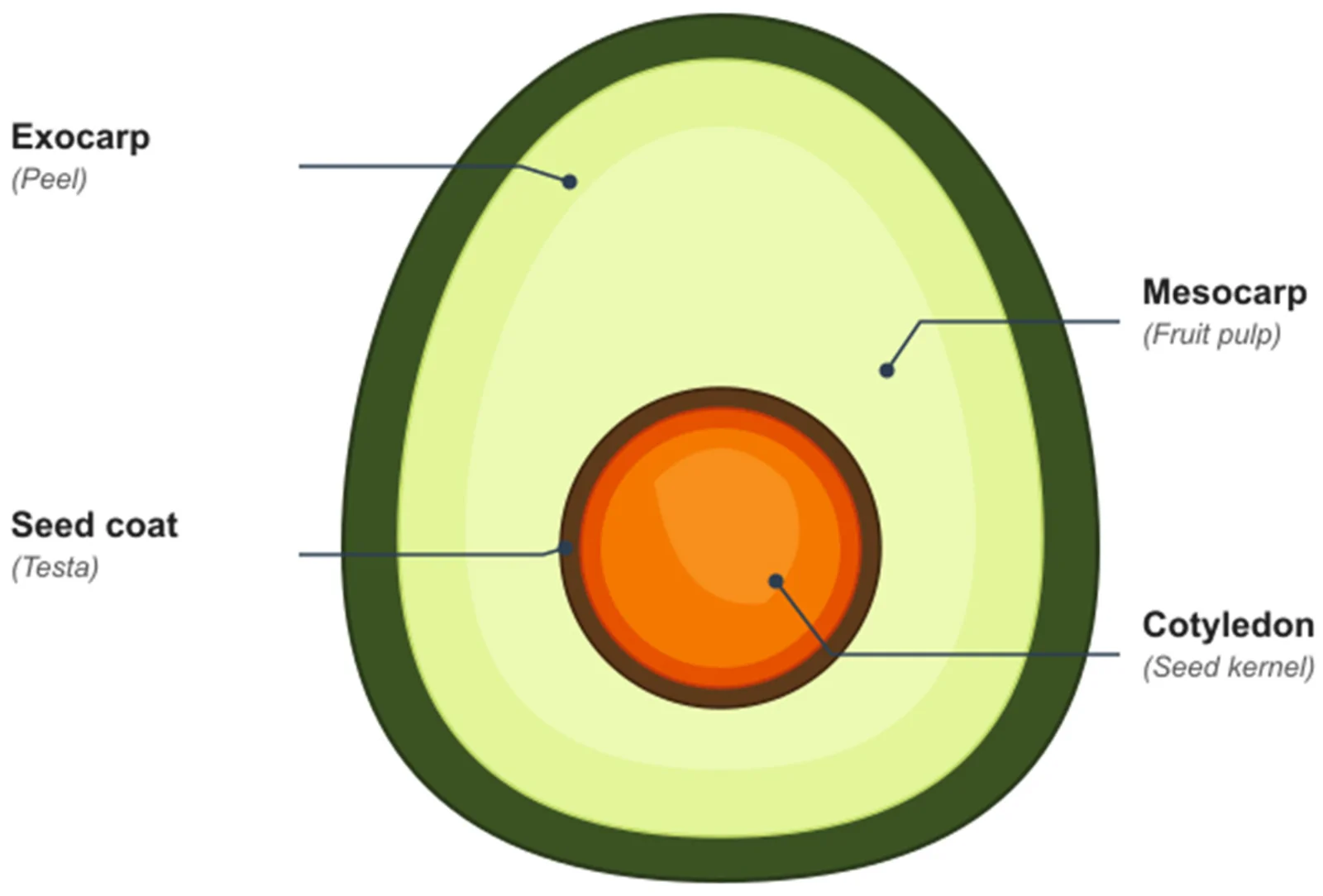
Schematic cross-section of avocado (*Persea americana* Mill.) fruit illustrating the four distinct anatomical fractions evaluated in this study: exocarp (peel), mesocarp (fruit pulp), seed coat (testa), and cotyledon (seed kernel).

**Table 1:** Yields and mass balance of dry extracts obtained from powdered avocado fruit fractions (*Persea americana* Mill.) using two extraction protocols.

Fruit Fraction (Anatomical Part)	Powdered Sample Yield (g/kg Fresh Fruit)	Protocol	Processed Sample (W_sample_, g)	Crude Extract (W_extract_, g)	Extraction Yield (%)
Exocarp (Peel)	170.57	CO_2_-Water	5.00	0.94	18.80
		Methanolic	10.00	2.41	24.10
Mesocarp (Fruit pulp)	715.31	CO_2_-Water	5.00	0.58	11.60
		Methanolic	10.00	0.83	8.30
Seed coat (Testa)	2.75	CO_2_-Water	5.00	1.15	23.00
		Methanolic	10.00	2.85	28.50
Cotyledon (Seed kernel)	111.37	CO_2_-Water	5.00	0.63	12.60
		Methanolic	10.00	1.52	15.20

**Table 2:** Total phenolic content (TPC, mg GAE/g dry extract) and total flavonoid content (TFC, mg QE/g dry extract) of different avocado fractions extracted with methanol and CO_2_-water.

MeOH Extracts	TPC mg GAE/g Dry Extract	TFC mg QE/g Dry Extract	CO_2_-Water Extracts	TPC mg GAE/g Dry Extract	TFC mg QE/g Dry Extract
fruit	65.129 ± 0.080 ^b^	32.352 ± 0.025 ^b^	fruit	31.546 ± 0.038 ^c^	2.940 ± 0.012 ^c^
peel	76.034 ± 0.086 ^a^	35.042 ± 0.026 ^a^	peel	41.381 ± 0.078 ^a^	4.962 ± 0.022 ^a^
seed	22.615 ± 0.019 ^d^	3.954 ± 0.006 ^d^	seed	20.022 ± 0.076 ^d^	2.213 ± 0.018 ^d^
seed coat	37.114 ± 0.083 ^c^	8.475 ± 0.019 ^c^	seed coat	33.990 ± 0.054 ^b^	3.367 ± 0.017 ^b^

Values are expressed as mean ± SD (*n* = 3). Different lowercase superscripts (a–d) within the same column indicate statistically significant differences among avocado fractions (*p* < 0.05, Tukey’s HSD test).

**Table 3:** DPPH radical scavenging activity, CUPRAC reducing power, and TEAC values of avocado fraction extracts.

Extraction	Fraction	DPPH (µmol TE/g Dry Extract)	CUPRAC (µmol TE/g Dry Extract)	TEAC (µmol/g Dry Extract)
MeOH	fruit	158.785 ± 0.190 ^a^	333.521 ± 2.102 ^b^	17.566 ± 0.006 ^b^
	peel	73.429 ± 0.116 ^c^	467.776 ± 1.657 ^a^	17.754 ± 0.005 ^a^
	seed	74.787 ± 0.180 ^b^	171.297 ± 0.762 ^c^	5.706 ± 0.001 ^d^
	seed coat	64.239 ± 0.330 ^d^	147.405 ± 1.177 ^d^	15.287 ± 0.002 ^c^
CO_2_-water	fruit	63.443 ± 0.060 ^b^	285.269 ± 0.844 ^b^	4.377 ± 0.003 ^b^
	peel	55.901 ± 0.148 ^c^	340.690 ± 0.697 ^a^	3.991 ± 0.003 ^c^
	seed	68.948 ± 1.133 ^a^	210.065 ± 0.648 ^c^	2.138 ± 0.002 ^d^
	seed coat	41.576 ± 0.198 ^d^	146.331 ± 2.237 ^d^	16.478 ± 0.006 ^a^

Values are expressed as mean ± SD (*n* = 3). Different lowercase superscripts (a–d) within the same column indicate statistically significant differences among avocado fractions (*p* < 0.05, Tukey’s HSD test).

**Table 5:** Validation parameters of the HPLC method for quantification of phenolic compounds.

Analyte	Retention Time (min)	Calibration Equation	R^2^	Tailing Factor	LOD (mg/mL) *	LOQ (mg/mL) *	Precision RSD (%) **
gallic acid	4.143	y = 2402.90304x − 4.50857	0.9989	0.942	0.015	0.045	1.414
chlorogenic acid	15.742	y = 629.31970x + 1.28641	0.9983	1.065	0.012	0.042	1.445
caffeic acid	17.556	y = 2102.65757x − 7.07645	0.9939	1.086	0.014	0.052	0.775
syringic acid	21.528	y = 86.58305x + 0.240058	0.9987	1.045	0.017	0.041	1.711
p-coumaric acid	22.489	y = 1689.65781x + 1.40244	0.9971	1.169	0.005	0.015	1.249
ferulic acid	25.458	y = 701.54315x + 1.67928	0.9989	1.201	0.018	0.047	1.215
*trans*-cinnamic acid	58.834	y = 29125.02593x − 7.45547	0.9983	1.147	0.006	0.018	1.615
quercetin	70.113	y = 3974.39145x − 3.5250	0.9990	1.354	0.016	0.044	1.654

* LOD and LOQ were calculated with *n* = 10; ** RSD was calculated with *n* = 6; RT = retention time, Figure S1.

**Table 6:** HPLC quantification of phenolic compounds in avocado extracts (mg/gram dry extract).

	MeOH				CO_2_-Water			
Analyte	Fruit	Peel	Seed	Seed Coat	Fruit	Peel	Seed	Seed Coat
gallic acid	2.236 ± 0.0056 ^a^	0.224 ± 0.0005 ^c^	0.318 ± 0.0030 ^b^	<LOD	0.036 ± 0.0002	ND	0.0153 ± 0.0006	<LOD
chlorogenic acid	4.216 ± 0.0015 ^a^	0.982 ± 0.0026 ^b^	0.565 ± 0.0040 ^c^	ND	0.0254 ± 0.0003	0.0300 ± 0.0000	<LOD	ND
caffeic acid	1.155 ± 0.0010 ^a^	0.202 ± 0.0006 ^c^	0.265 ± 0.0000 ^b^	ND	ND	<LOD	0.0257 ± 0.0006	ND
syringic acid	0.954 ± 0.0021 ^a^	0.104 ± 0.0021 ^c^	0.166 ± 0.0015 ^b^	ND	ND	ND	ND	ND
p-coumaric acid	1.025 ± 0.0014 ^a^	0.198 ± 0.0012 ^b^	0.096 ± 0.0010 ^c^	ND	ND	ND	ND	ND
ferulic acid	2.656 ± 0.0026 ^a^	0.978 ± 0.0145 ^b^	0.105 ± 0.0030 ^c^	ND	0.104 ± 0.0010	<LOD	ND	ND
*trans*-cinnamic acid	1.247 ± 0.0015 ^a^	0.653 ± 0.0017 ^b^	0.361 ± 0.0055 ^c^	ND	ND	ND	ND	ND
quercetin	0.523 ± 0.0006 ^a^	0.106 ± 0.0010 ^c^	0.109 ± 0.0015 ^b^	0.026 ± 0.0006 ^d^	ND	ND	ND	ND

The results are expressed in mean ± standard deviation with their respective units (*n* = 3). Different lowercase letters within the same row indicate statistically significant differences among fractions (Tukey’s HSD, *p* < 0.05). ND: not detected; <LOD: below the limit of detection.

**Table 7:** IC_50_ values for α-amylase and β-glucosidase inhibition of avocado extracts and acarbose (positive control) (mg/mL).

Extraction	Fraction	α-Amylase IC_50_ (mg/mL)	β-Glucosidase IC_50_ (mg/mL)
MeOH	fruit	1.84	2.35
	peel	1.55	2.20
	seed	1.38	2.25
	seed coat	1.25	1.89
CO_2_-water	fruit	1.95	2.98
	peel	1.86	2.75
	seed	1.75	2.65
	seed coat	1.65	2.05
* Acarbose (positive control) *	—	0.86	1.18

* Acarbose was used as positive control for both enzymes.

**Table 8:** Technical parameters and method parameters of the applied extraction protocols.

Extraction Parameter	CO_2_-Assisted Aqueous Extraction (CO_2_-Water)	Methanolic Extraction
Reference Method	[22]	[39]
Sample Amount (Wsample)	5.0 g	10.0 g
Solvent Type	CO_2_-saturated ultrapure water (pH 3.7–4.5)	100% HPLC-grade Methanol
Solvent Volume	50 mL	50 mL
Solid-to-Solvent Ratio (*w*/*v*)	1:10 g/mL	1:5 g/mL
Pre-treatment/Cell Disruption	Sonication (3 × 1 min, 35 kHz)	None
Extraction Conditions	Orbital shaking (55 °C, 1 h)	Orbital shaking (40 °C, 8 h)
Post-Extraction Handling	+4 °C overnight passive extraction	Centrifugation (5000 rpm, 20 min)
Separation Method	Filtration (Whatman No. 1)	Vacuum filtration + Centrifugation
Solvent Removal	Rotary evaporation (40 °C)	Rotary evaporation (40 °C)

## Data Availability

The original contributions presented in this study are included in the article/Supplementary Material. Further inquiries can be directed to the corresponding author.

## Supplementary Materials

The following supporting information can be downloaded at: https://www.mdpi.com/article/10.3390/molecules31193418/s1. Figure S1. HPLC-UV chromatograms and calibration equations of the phenolic compound standard mixture (mix5) recorded at 280 nm (MWD1 A) and 360 nm (MWD1 C). Peaks identified: gallic acid, chlorogenic acid, caffeic acid, syringic acid, p-coumaric acid, ferulic acid, trans-sinnamic acid and quercetin.

## Abbreviations

TPC	Total phenolic content
TFC	Total flavonoid content
DPPH	2,2-diphenyl-1-picrylhydrazyl
CUPRAC	Cupric Ion-Reducing Antioxidant Capacity
TEAC	Trolox Equivalent Antioxidant Capacity
HPLC	High-Performance Liquid Chromatography
PCA	Principal Component Analysis
HCA	Hierarchical Cluster Analysis
