# Supplementary material for: Phytochemical Profiling, Antioxidant Capacity, and In Vitro α-Amylase/β-Glucosidase Inhibitory Activity of Different Avocado Tissues Under Methanolic and CO2-Assisted Aqueous Extraction
Source: Molecules. 2026 Sep 25;31(19):3418. doi: 10.3390/molecules31193418 (PMC13666510; doi:10.3390/molecules31193418)
Supplement: Supplementary file 1 [file molecules-31-03418-s001.zip › molecules-4507251-supplementary.pdf]

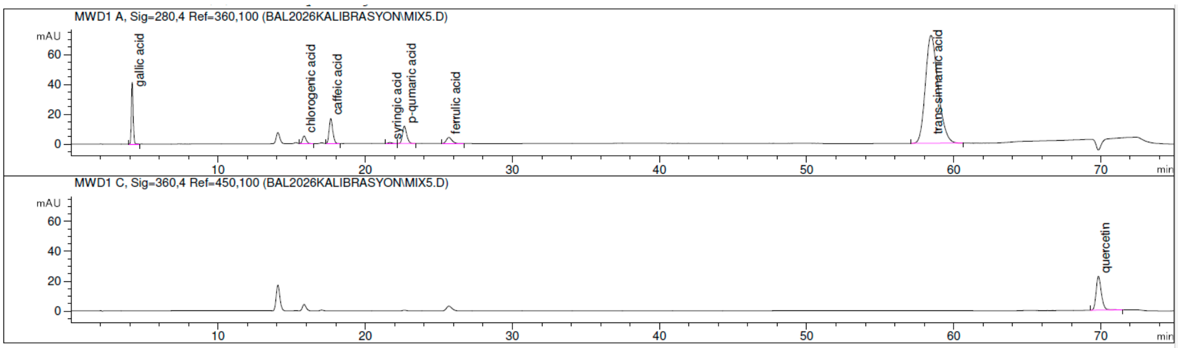

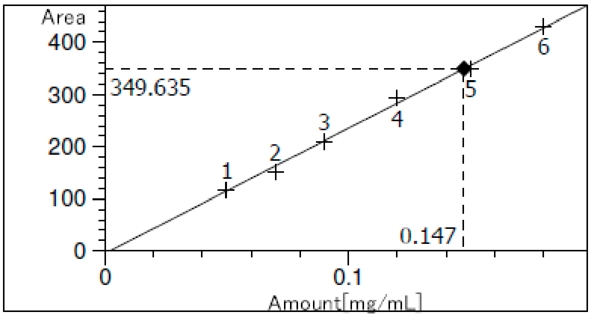

gallic acid at exp. RT: 4.143
MWD1 A, Sig=280,4 Ref=360,100
Correlation: 0.99887
Residual Std. Dev.: 7.68903
Formula: y = mx + b
m: 2402.90304
b: -4.50857
x: Amount
y: Area

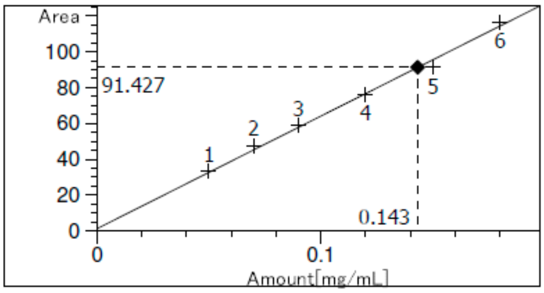

chlorogenic acid at exp. RT: 15.742
MWD1 A, Sig=280,4 Ref=360,100
Correlation: 0.99826
Residual Std. Dev.: 2.49516
Formula: y = mx + b
m: 629.31970
b: 1.28641
x: Amount
y: Area

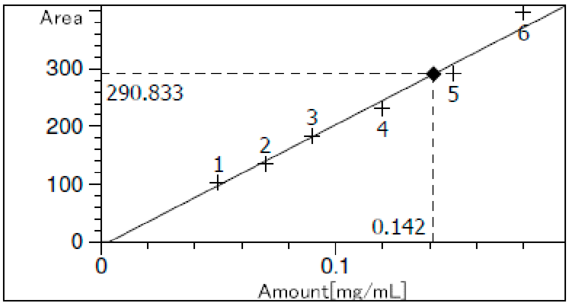

caffeic acid at exp. RT: 17.556
MWD1 A, Sig=280,4 Ref=360,100
Correlation: 0.99388
Residual Std. Dev.: 15.70762
Formula: y = mx + b
m: 2102.65757
b: -7.07645
x: Amount
y: Area

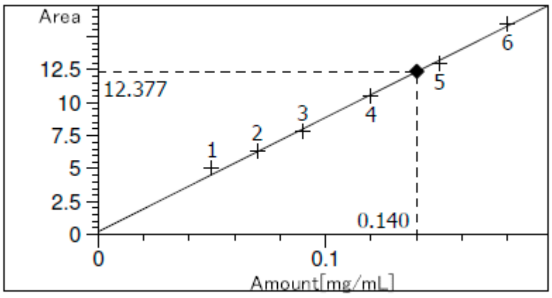

syringic acid at exp. RT: 21.528
MWD1 A, Sig=280,4 Ref=360,100
Correlation: 0.99869
Residual Std. Dev.: 0.29788
Formula: y = mx + b
m: 86.58305
b: 2.40058e-1
x: Amount
y: Area

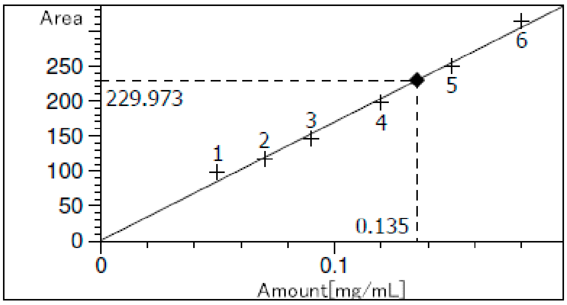


```
p-qumaric acid at exp. RT: 22.489
MWD1 A, Sig=280,4 Ref=360,100
Correlation:            0.99707
Residual Std. Dev.:     8.70586
Formula: y = mx + b
        m:   1689.65781
        b:      1.40244
        x: Amount
        y: Area
```

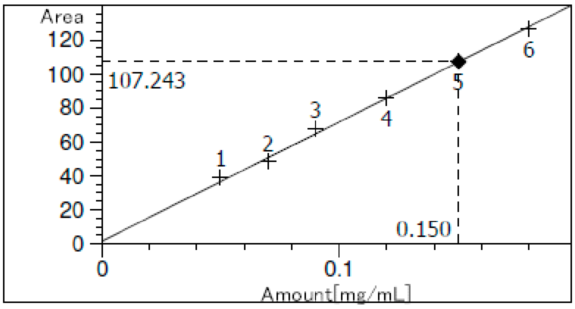


```
ferrulic acid at exp. RT: 25.458
MWD1 A, Sig=280,4 Ref=360,100
Correlation:            0.99888
Residual Std. Dev.:     2.23234
Formula: y = mx + b
        m:    701.54315
        b:      1.67928
        x: Amount
        y: Area
```

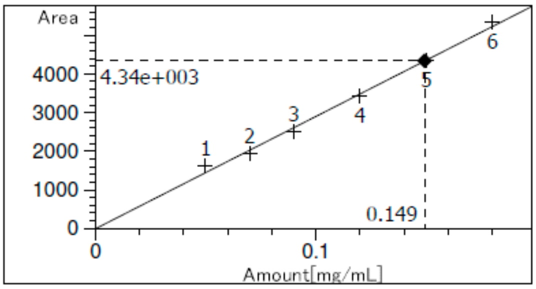


```
trans-sinnamic acid at exp. RT: 58.834
MWD1 A, Sig=280,4 Ref=360,100
Correlation:            0.99834
Residual Std. Dev.:   112.79121
Formula: y = mx + b
        m:  29125.02593
        b:     -7.45547
        x: Amount
        y: Area
```

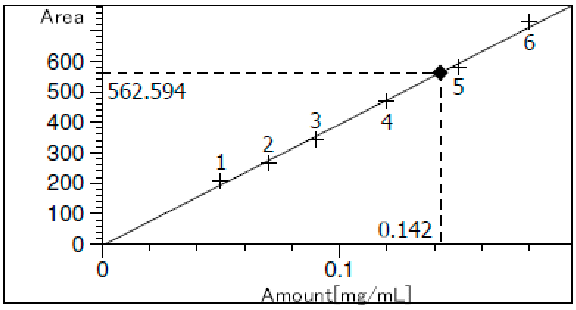


```
quercetin at exp. RT: 70.113
MWD1 C, Sig=360,4 Ref=450,100
Correlation:            0.99897
Residual Std. Dev.:    12.10806
Formula: y = mx + b
        m:   3974.39145
        b:     -3.52499
        x: Amount
        y: Area
```

Figure S1. HPLC-UV chromatograms and calibration equations of the phenolic compound standard mixture (mix5) recorded at 280 nm (MWD1 A) and 360 nm (MWD1 C). Peaks identified: gallic acid, chlorogenic acid, caffeic acid, syringic acid, p-coumaric acid, ferulic acid, trans-sinnamic acid and quercetin.
